# Successful management of massive digoxin overdose using DIGIFab and therapeutic plasma exchange: a case report

**DOI:** 10.1186/s13256-024-04386-6

**Published:** 2024-03-05

**Authors:** Reema M. Alhussein, Nawaf A. Alamri, Hussain M. Alhashem, Mohammed I. Alarifi, Bader Alyahya

**Affiliations:** 1 Department of Emergency Medicine, King Saud University, Medical City, Riyadh, Saudi Arabia; 2 Department of Critical Care Medicine, College of Medicine, King Saud University, Riyadh, Saudi Arabia; 3 Department of Emergency Medicine, College of Medicine, King Saud University, Riyadh, Saudi Arabia

**Keywords:** Digoxin, Plasma exchange, TPE, DIGIFab, Drug overdose, ECTR, Case report

## Abstract

**Background:**

Despite the efficacy and safety of DIGIFab, it is relatively expensive and has limited availability. In addition, alternative interventions, such as therapeutic plasma exchange, may need to be considered in massive digoxin overdoses. Although few case reports describe its efficacy.

**Case presentation:**

We report a case of a 17-year-old white male patient brought by family members to our emergency department in Riyadh, Saudi Arabia. After intentionally ingesting 48 mg of digoxin tablets to commit suicide, the patient’s initial digoxin serum level was 8.04 ng/mL. The patient was resuscitated in the emergency department. After admission to the intensive care unit, the patient underwent therapeutic plasma exchange, because of insufficient DIGIFab doses. Afterward, the serum digoxin levels drastically decreased, and his symptoms reverted. The patient was successfully managed and discharged 7 days after admission.

**Conclusion:**

Despite insufficient evidence and a limited number of case reports describing the use of extracorporeal treatment in digoxin overdose, we noted the significant impact of therapeutic plasma exchange on our patient. However, therapeutic plasma exchange’s use in routine treatment requires stronger evidence to confirm its benefits.

## Background

Digoxin has positive inotropic and negative chronotropic effects on the heart, and is primarily used in treating heart failure and tachydysrhythmias [1]. In clinical practice, there is a high probability of accidental intoxication with digitalis owing to the narrow therapeutic window [2]. However, the number of reported suicide cases using digoxin was significant [3–6]. Compared with other medications, massive digoxin intoxication after a suicide attempt is uncommon [1].

Management of digitalis intoxication can be challenging. Digoxin-specific antibody fragments (DIGIFab) are indicated in cases with severe symptoms or in cases of massive overdose. It is the definitive treatment for life-threatening digitalis toxicity [7]. There are other methods to accelerate the clearance of drugs from the body, including therapeutic plasma exchange (TPE); although it is unable to clear digoxin, it has shown some success, mainly in cases of renal failure [8]. Furthermore, in one study, hemoperfusion did not significantly improve digoxin clearance because of digoxin’s large distribution volume [9]. Here, we present a case that illustrates the successful use of TPE after an insufficient DIGIFab dose to treat intoxication caused by the intentional ingestion of 192 tablets of 0.25 mg digoxin in an otherwise healthy 17-year-old patient. To date, only a few case reports have described the use of such a therapeutic approach in healthy patients with digoxin overdose.

## Case presentation

**Fig. 1:**
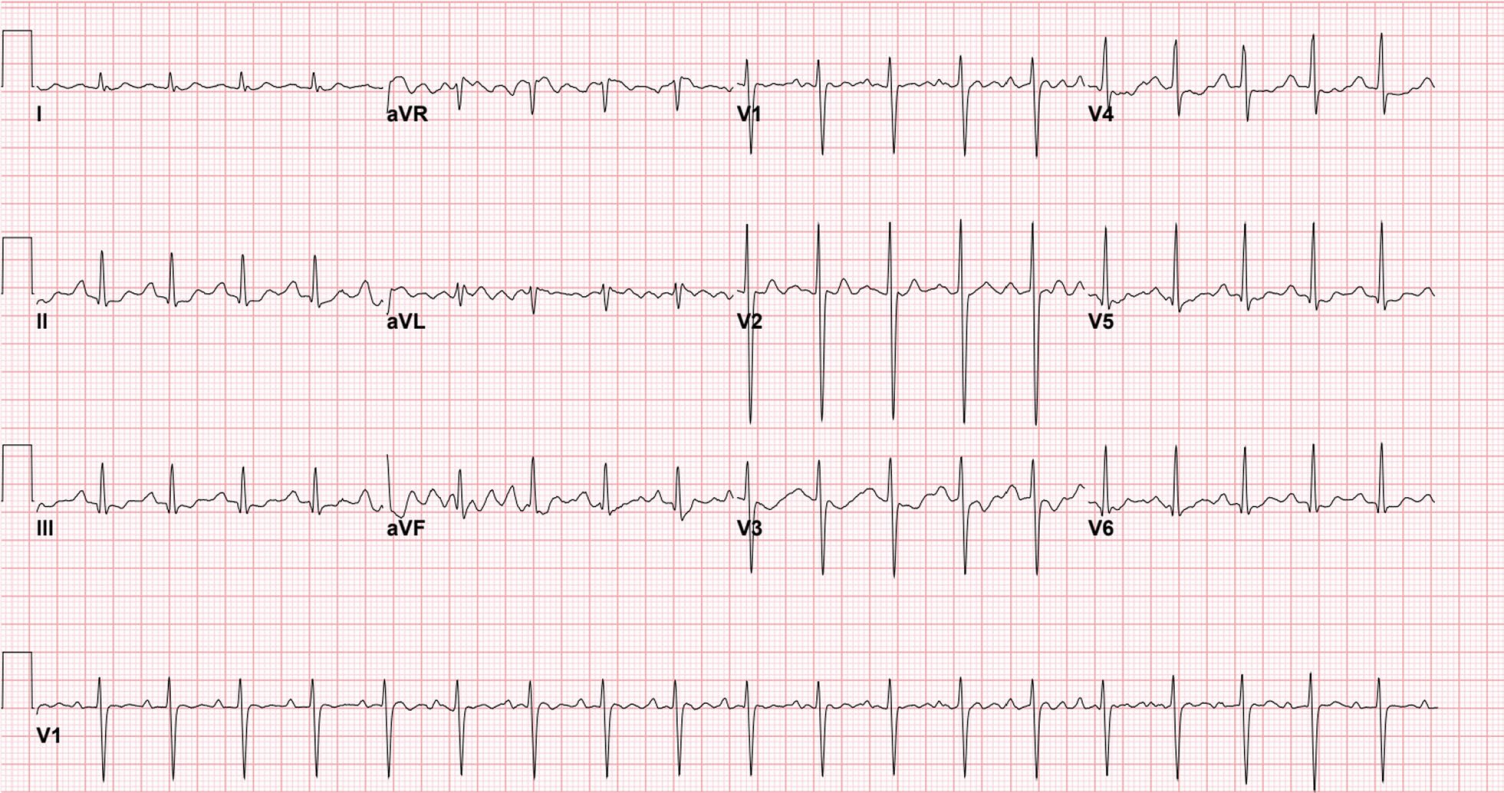
Initial electrocardiogram revealed a sinus tachycardia

**Fig. 2:**
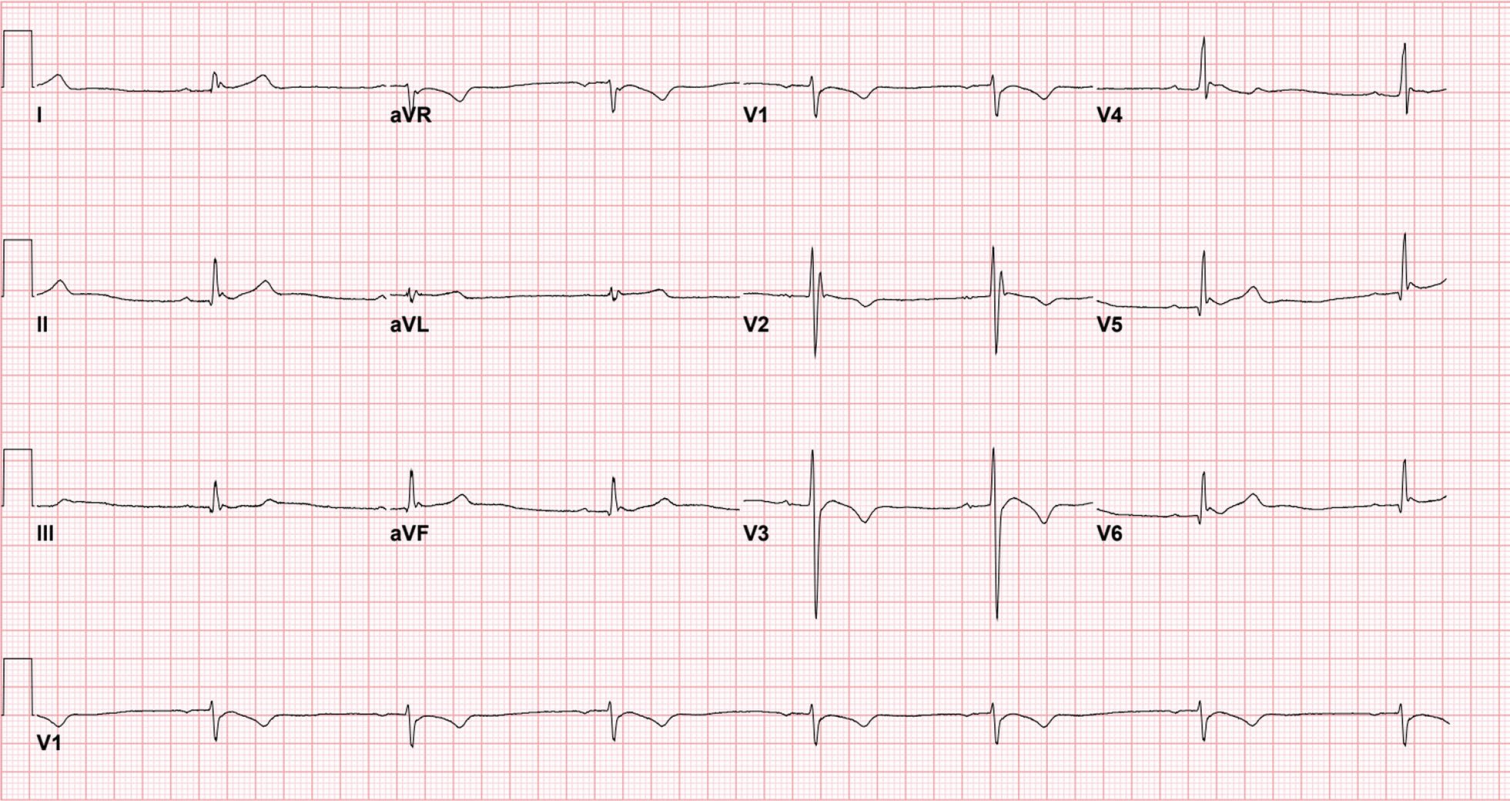
Electrocardiogram revealed a sinus bradycardia, first-degree heart block with T-wave inversion in anterio-septal leads

**Fig. 3:**
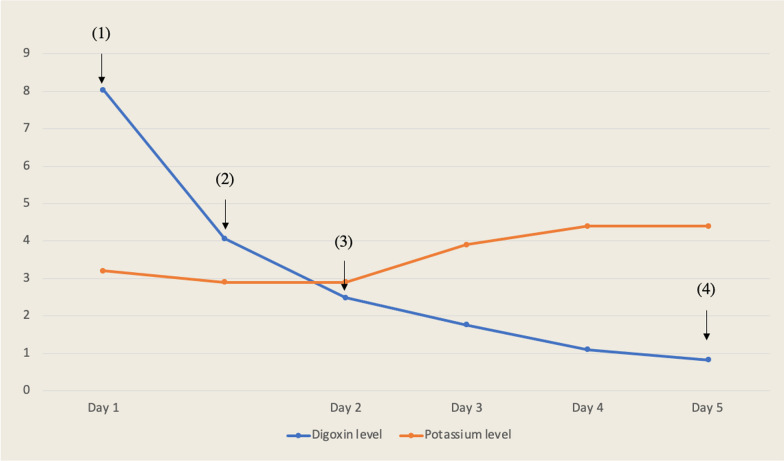
Our patient’s serum digoxin and potassium concentrations during and since presentation. Arrow (1): Initial digoxin level upon arrival 4 hours post-ingestion. Arrow (2): Digoxin level after therapeutic plasma exchange and DIGIFab. Arrow (3): Serum digoxin on second day. Arrow (4): Upon discharge

The patient was a 17-year-old white male, previously in good health. He was not on any medications, he was a non-smoker, and he weighed 50 kg. Regarding the social status of the patient, he was living with his family, as he was high school student. He was brought by family members to our emergency department (ED) in Riyadh, Saudi Arabia. The patient was admitted after an overdose of digoxin 4 hours before presentation in a suicide attempt. He ingested 192 tablets of 0.25 mg digoxin (total dose of 48 mg). He developed dizziness, nausea, and vomiting after ingestion. Upon presentation, his Glasgow Coma Scale (GCS) score was 14/15, as the patient’s eyes opened to verbal commands. He was slightly drowsy but responded to questions and was fully oriented. The rest of other systems examinations were all unremarkable. The patient’s vital signs were as follows: heart rate, 118/minute; blood pressure (BP), 128/90 mmHg; respiratory rate, 19 breaths/minute; and maintaining oxygen saturation, 99% on room air. The initial electrocardiogram (ECG) revealed sinus tachycardia (Fig. 1). While the patient was being assessed, he started having more symptoms, including seeing yellow hues and having a decrease in consciousness level, with a GCS score of 8/15. He responded with incomprehensible sounds, withdrawing his limbs and opening his eyes to pain. As the patient’s GCS score deteriorated, he was intubated for airway protection. After securing the patient’s airway, 50 g of activated charcoal was administered through a nasogastric tube (NGT). Shortly thereafter, despite the patient being under sedation using propofol and fentanyl infusions, he showed synchronized tonic–clonic movement of all limbs, for which he received midazolam 5 mg. Five DIGIFab vials (200 mg) were administered as a bolus after extracting all routine laboratory tests, along with serum digoxin levels and urine toxicology screening. The intensive care unit (ICU), toxicology center, and cardiology services were consulted. He was immediately transferred to the ICU for further medical management. The initial serum chemistry was normal, except for a potassium level of 2.9 mmol/L, so he received 20 mmol of potassium chloride intravenously. The toxicological levels in our patient revealed a digoxin level of 8.04 ng/mL in the blood 4 hours post-ingestion and before he received the DIGIFab. Labs results, including complete blood count (CBC) and renal and liver functions, were all within normal range. In addition, serum ethanol concentration and urine toxicology screening results were negative for cocaine, opioids, cannabis, and amphetamine. In the ICU, the patient received five vials of DIGIFab intravenously. Given the high serum digoxin level and the patient’s condition, both a dialysis line and a central venous catheter (CVC) were inserted electively, and were used later for TPE management and medication administration, respectively. TPE was performed for over 2 hours using a dual-lumen CVC, (replacement used = 2210 ml; plasma removed = 2345 ml; TBV processed = 1.1 volume). Serum digoxin measured immediately after TPE was 4.07 ng/ mL (Fig. 3). Shortly after TPE, the patient’s ECG demonstrated a first-degree heart block (Fig. 2) with a heart rate of 43 beats per minute (BPM) and T-wave inversion. Another two vials (80 mg) of DIGIFab were used. The patient was closely monitored, and serum electrolyte and digoxin levels were regularly monitored, showing a gradual decrease over time (Fig. 3). Four days after continuous monitoring, gradual improvement was marked clinically, with the absence of any ECG changes and the disappearance of any signs of digoxin overdose. Two days later, the patient was extubated and found to have hyperactive delirium with agitation; he was disoriented and was pulling on his lines. Haloperidol (5 mg) was administered. Subsequently, the patient gradually became less agitated and showed an improvement in his mental status. The following day, the patient’s mental status returned to baseline, and he was transferred to a general ward, where he underwent a psychiatric assessment and was discharged in a stable condition after 7 days of hospitalization. Upon follow-up in 6 months, a phone call was made to his legal guardian to check up on the patient, since the discharging team did not schedule any follow-up as the patient status was not needed. According to him, he is in good health and doing regular follow-up with his primary care and psychiatric physicians.

## Discussion

Our article reports a case of a young and previously healthy male who ingested 192 tablets of 0.25 mg digoxin, and underwent timely and aggressive management, including immediate resuscitation, activated charcoal, DIGIFab, and TPE. This helped the patient to return back to his regular life completely healthy after 7 days of hospitalization.

Understanding the pharmacological actions of digoxin will help explain its toxic effects. It has a weak positive inotropic effect, by inhibiting the Na^+^–K^+^ ATPase pump and indirectly increasing calcium availability to the contractile elements of the myofibril [10]. Although toxicity is infrequent, it has serious clinical manifestations [11]. The case presented in this report emphasizes the different digoxin toxicity presentations and the significance of initiating aggressive and early management and considering other modalities of treatment based not only on serum drug levels but also on clinical presentation. Due to early initiation of DIGIFab, TPE, and activated charcoal, the patient showed a successful gradual response, with digoxin levels quickly declining and dramatic improvement in his clinical manifestations.

Digoxin toxicity presents nonspecifically as cardiac or non-cardiac symptoms, making diagnosis difficult [10, 12–14]. Gastrointestinal symptoms are the most common presentation, accounting for 30–70% of cases [15–17]. Cardiotoxic effects of digoxin can manifest as arrhythmias and conduction disturbances, but the frequency of these is controversial [18]. Early dysrhythmias associated with digoxin toxicity are premature ventricular contractions (PVC),

Conduction blocks in the intoxication setting can range from first-degree atrioventricular (AV) block to complete heart block, with a low incidence of sinus bradycardia and tachycardia [19]. Neuropsychiatric presentations are less common and often attributed to other causes. Few cases have reported lethargy, delirium, seizures, and visual disturbances as side effects of intoxication. Digoxin-induced seizures exhibit chronic toxicity, with frequent attacks showing metabolic and epileptiform patterns on EEG. These seizures resolve completely with digoxin toxicity treatment [20].

Digoxin toxicity is classically associated with significant hyperkalemia [21]; however, in our case, the patient had an initial potassium level of 2.9 mmol/L even before initiating the management, which goes against the classical finding of hyperkalemia reported in the literature. This is an interesting paradoxical finding, as there was no acute co-ingestion of other medications, and our patient was a healthy young male not taking any medications or supplements.

Specific therapies for digoxin toxicity have aimed at rapidly reducing the serum digoxin concentration, including the administration of DIGIFab, as it binds to digoxin, forming a complex excreted in the urine. However, despite the efficacy and safety of DIGIFab, it is expensive, and availability is limited; thus, alternative measures may need to be considered for severe intoxication [11]. Administration of DIGIFab should be based on the serum concentration or history and clinical state of the patient, supported by biological monitoring. If the clinical response after administration is not seen within 2 hours, a further dosage should be administered [20]. Using the DIGIFab dose calculation [22], total body load was calculated by multiplying the dose of digoxin ingested (48 mg) by 0.8 (digoxin bioavailability), and the total body load of digoxin was divided by the amount of digoxin neutralized per vial (0.5 mg per vial; DIGIFab dose (number of vials) = total digoxin load (mg)/0.5 mg of digoxin neutralized per vial). Based on the ingestion amount, our patient’s calculated dose was 76.8 vials. This amount was not available at our center during the initial resuscitation of the patient.

Different ECTR modalities have been used to treat digoxin toxicity. TPE and other ECTR have been suggested as possible interventions in patients with massive overdose, especially when DIGIFab is unavailable, or doses are insufficient. TPE has been shown to be potentially effective, as it removes toxic substance rapidly from the blood stream, allowing removal of protein-bound molecules and large molecular weight [23]. In our case, the choice to treat the patient with TPE was mainly driven by the insufficient availability of DIGIFab vials. However, we observed a significant improvement in patient manifestations and a reduction in digoxin serum levels (Fig. 3). However, the level of evidence was determined to be low, as the vast majority of available data evaluating the effectiveness of ECTR in digoxin poisoning in both acute and chronic ingestion are case reports and case series with no controlled trials allowing adequate comparison of the risks and benefits of TPE in digoxin toxicity [8, 9, 24, 25]. It has been suggested that starting a TPE session up to 3 hours after administration of DIGIFab will possibly maximize Fab–digoxin clearance [8]. In one study [26], a TPE removed 0.250 mg over a 90-minute session successfully, which might be explained by modifications of digoxin toxicokinetics following Fab administration. An experimental study [27] has examined the effects of both hemodialysis and plasmapheresis after induced digoxin intoxication, with the latter being more effective. However, the efficacy was mainly dependent on the plasma exchange rate. In all experiments with either hemodialysis or plasmapheresis, the levels increased again to some extent after cessation of the dialysis, which probably related to the release of the drug from tissue storage. Thus, for the effective elimination of the drug, it might be necessary to perform repetitive plasmapheresis for a certain period of time.

In our patient experience, administration of DIGIFab followed closely by a 2-hour TPE single session was probably the key of treatment efficacy.

## Conclusion

Patients’ clinical response to the initial dose of DIGIFab serves as a guide for the need for further doses and the use of other adjunctive modalities, such as TPE. However, despite insufficient evidence, unavailability of clinical trials, and a limited number of case reports describing the use of ECTR in digoxin overdose, we noticed a significant effect of TPE on clinical symptoms and digoxin serum levels. However, the use of this modality for routine treatment requires stronger evidence to establish its benefits, a further protocols that can help us understand when and under what circumstances TPE can be beneficial for patients with digoxin toxicity.

## Data Availability

The data used to support the findings of this study are available from the corresponding author upon request.

## Abbreviations

TPE: Therapeutic plasma exchange
ICU: Intensive care unit
ED: Emergency department
ECTR: Extracorporeal treatment
CVC: Central venous catheter
GCS: Glasgow coma scale
